# Stripping off the rice panicle: induced genetic variation awakens the sheathed spikelet for a better yield

**DOI:** 10.1093/jxb/erae327

**Published:** 2024-09-26

**Authors:** Jitendra K Mohanty, Swarup K Parida

**Affiliations:** National Institute of Plant Genome Research (NIPGR), Aruna Asaf Ali Marg, New Delhi 110067, India; National Institute of Plant Genome Research (NIPGR), Aruna Asaf Ali Marg, New Delhi 110067, India

**Keywords:** CPE, mutagenesis, panicle, QTL, rice

## Abstract

This article comments on:

**Ballichatla S, Gokulan CG, Barbadikar KM, Hake AA, Potupureddi G, Guha PK, Phule AS, Magar ND, Balija V, Awalellu K, Kokku P, Golla S, Sundaram RM, Padmakumari AP, Laha GS, Senguttuvel P, Lella Venkata SR, Patel HK, Sonti RV, Maganti SM.** 2024. Impairment in a member of AP2/ERF and F-box family protein enhances complete panicle exsertion in rice. Journal of Experimental Botany **75**, https://doi.org/10.1093/jxb/erae244.

This article comments on:

**Ballichatla S, Gokulan CG, Barbadikar KM, Hake AA, Potupureddi G, Guha PK, Phule AS, Magar ND, Balija V, Awalellu K, Kokku P, Golla S, Sundaram RM, Padmakumari AP, Laha GS, Senguttuvel P, Lella Venkata SR, Patel HK, Sonti RV, Maganti SM.** 2024. Impairment in a member of AP2/ERF and F-box family protein enhances complete panicle exsertion in rice. Journal of Experimental Botany **75**, https://doi.org/10.1093/jxb/erae244.

**The rice panicle is the reproductive structure that contains the seeds, so a productive spikelet in a panicle contributes to increased yield. A sheathed panicle covers a significant portion of spikelets with the flag leaf and impairs effective pollination which subsequently compromises the yield. Through induced mutagenesis and integrated molecular breeding strategies, Ballichatla *et al*. (2024) uncovered the intricate regulation of complete panicle exsertion (CPE) in rice. This study also delineates a set of high-confidence novel genomic loci and superior gene alleles which have a substantial translational significance in rice crop improvement towards future food security.**

## Complete panicle exsertion: tailoring the rice panicle architecture for agronomic and economic advantage

**Fig. 1.**
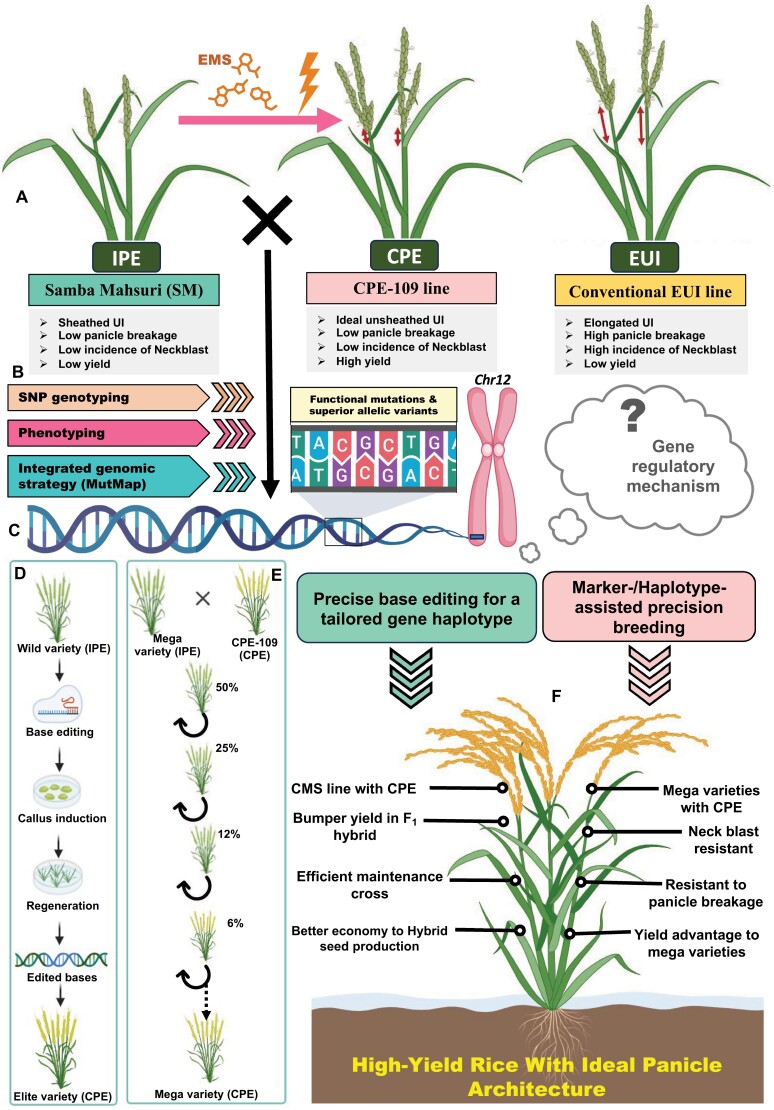
Induced mutagenesis-based genomic strategy to dissect the complete panicle exsertion (CPE) trait for rice crop improvement. (A) Schematic representation of different panicle architectures, such as incomplete panicle exsertion (IPE), CPE, elongated uppermost internode (EUI), and their associated concerns in rice crop production. Ballichatla *et al*. (2024) exposed the IPE-type Samba Mahsuri (SM) to EMS-induced mutagenesis to develop a CPE-109 line, which was subsequently crossed with the parental SM to generate a mapping population. (B) Large-scale phenotyping and genome-wide SNP genotyping deployed in an integrated genomic strategy (including MutMap) delineated potential genomic regions controlling the CPE trait in rice. (C) The delineated functional mutations and the allelic variants regulate CPE by modulating several hormonal biosynthetic process; however, the intricate gene regulatory mechanism is yet to be deciphered. The identified functional loci have a great significance in translational genomics (genome editing and marker-assisted breeding) for rice improvement targeting the CPE trait. (D) Precise/targeted editing of the genetic background of wild-type landraces (IPE) to develop elite varieties with CPE. (E) Marker-/haplotype-assisted introgression breeding to improve the existing mega rice varieties with the CPE trait. (F) Induced mutagenesis-based integrated genomic strategy led to development of high-yielding rice varieties restructured with desirable panicle architecture (CPE) which has high agronomical and economic importance. The image of the crops are created with Biorender.com.

The panicle architecture plays a vital role in determining the yield and productivity of rice. Panicle exsertion is one such quantitative architectural trait, which is defined as the distance between the neck node of the panicle and the leaf cushion of the flag leaf in rice. CPE out of the flag leaf results in an unsheathed panicle with more reproductive success, whereas impaired panicle exsertion results in a sheathed panicle, affecting grain filling and subsequently crop yield in rice (Ma *et al*., 2002; Cruz *et al*., 2008; Liu *et al*., 2008). Although the phenomenon of panicle exsertion is observed to varying degrees in diverse germplasm accessions of rice, cytoplasmic male sterile (CMS) lines more frequently exhibit incomplete panicle exsertion (IPE). The spikelets in the partially enclosed panicle of CMS lines are impaired in effective pollination, leading to low fertilization and seed setting efficiency, which consequently causes a significant loss in rice seed yield (Shen *et al*., 1987; Yang *et al*., 2002; Yin *et al*., 2007; Cruz *et al*., 2008; Guan *et al*., 2011; Luo *et al*., 2013). Thus, the issue of IPE poses a significant threat to grain yield in general and to hybrid rice seed production in particular. This highlights the importance of CPE as a trait of both agronomic and economic value in the global rice trade and commerce. A pioneering effort to tackle this issue in rice has identified a phenotypic modification distinct from CPE, known as elongated uppermost internode (EUI), which has been shown to enhance panicle exsertion by elongating the uppermost internodes (Fig. 1A). Few forward genetics strategies have identified major quantitative trait loci (QTLs) and causal genes that govern the complex quantitative traits of EUI by modulating the gibberellin (GA) biosynthetic pathways. (Zhu *et al*., 2011; Ji *et al*., 2014; Gao *et al*., 2016; Zhan *et al*., 2019). However, these findings have not yet been translated efficiently to develop rice lines with desirable CPE phenotypes. Further, the EUI as compared with the CPE phenotype is associated with various adverse agronomic implications such as panicle breakage and a higher incidence of neck blast disease, thus affecting the overall grain yield in rice (Kalia and Rathour, 2019). Identifying the molecular genetic basis of CPE with limited internode elongation and fewer agronomic adversities is essential. However, the limited availability of natural accessions and breeding lines with contrasting CPE traits, along with the lack of genetic markers tightly linked to QTLs and genes governing CPE, hinders the genomics-assisted improvement of high-yield rice varieties. (Suneel *et al*., 2020). In this context, utilization of induced mutagenesis-mediated forward genetics strategies for generating novel functional mutations controlling agronomically important traits appears to be an attractive solution. This approach facilitates the quantitative dissection of the complex CPE trait and accelerates the genetic enhancement of rice.

## Induced mutagenesis: delineating untapped causative functional mutations to decipher the complex genetic architecture of CPE in rice

Induced mutagenesis for breeding, trait dissection, and crop genetic improvement is a widely accepted strategy that creates heritable genetic variations. It is particularly useful in cases where naturally occurring crop germplasm accessions lack sufficient trait variations. This is a well-established strategy that is used to identify novel mutations, genes, alleles, and gene functions of agronomic importance (Jankowicz-Cieslak *et al*., 2017). The current study by Ballichatla *et al*. (2024) along with their previous findings (Suneel *et al*., 2020; Potupureddi *et al*., 2021) utilized the power of the ethylmethane sulfonate (EMS)-induced mutagenesis strategy to develop a novel rice mutant (CPE-109) with CPE phenotype in the genetic background of a popular mega rice variety Samba Mahsuri (SM) (Fig. 1A). The mutant CPE-109 showed high genetic similarity as well as distinctness, uniformity, and stability (DUS) trait homology with its SM parent, except for the CPE trait. This suggests that CPE-109 can be directly introduced into the marker-assisted varietal breeding trial to develop an improved SM rice variety with a completely unsheathed panicle architecture and enhanced grain yield.

To accomplish this, the authors primarily deployed combinatorial molecular breeding and functional genomic strategies by integrating the traditional linkage (QTL) mapping and next-generation sequencing (NGS)-based bulk segregant (QTL-seq) assay with differential gene expression profiling (Fig. 1B). Briefly, a divergent RPHR-1005 line and wild-type SM displaying IPE were inter-crossed independently with CPE-109 to generate two different F_2_ mapping populations (i.e. CPE-109×RPHR-1005 and CPE-109×SM). The F_1_ plants from both of the mapping population crosses exhibit an IPE phenotype, indicating CPE as a recessive trait in rice. Continuous normal frequency distribution of exserted panicle length in the F_2_ individuals from both mapping populations highlights the quantitative trait genetic inheritance pattern of CPE. Further, QTL mapping and QTL-seq assays were deployed in the two mapping populations, and two overlapping major QTL genomic regions on chromosomes (Chr) 4 and 12 were deciphered that govern CPE in rice.

An extensive integrated genomic analysis identified two distinct single nucleotide polymorphisms (SNPs): KASP 1-12 (T→C; located at Chr 12:1269983) and KASP 12-16 (G→A; located at Chr 12:1515198) (Fig. 1C). These SNPs, found in the genes *Os12g0126300* and *Os12g0131400*, respectively, explained 81% and 60% of the phenotypic trait variation for CPE. The SNP KASP 12-12 (T→C), identified in the sixth exon of the AP2/ethylene-responsive transcription factor (*AP2*/*ERF*) gene (*Os12g0126300*), resulted in a missense mutation, changing methionine to valine at the 385th amino acid. In contrast, the SNP KASP 12-16 (G→A), located in the third exon of the F-box protein-coding gene (*Os12g0131400*), led to a nonsense mutation, causing premature termination at the 183rd amino acid. Differential gene expression profiling between CPE-109 and SM showed down-regulation of *AP2*/*ERF* in the CPE-109 mutant. This down-regulation may be due to an altered SNP (KASP 12-17) in the target gene promoter sequence, which co-segregates with the CPE trait in rice. Hence, the upstream regulatory mutation in the promoter, along with the missense mutations in coding regions of the *AP2/ERF* gene, can lead to reduced expression and functionality of this gene, respectively. This compromise in transcript abundance and functionality of the *AP2/ERF* gene is subsequently reported to down-regulate the ethylene biosynthesis process and up-regulate the GA and cytokinin biosynthesis cascades relaying the CPE phenotype. Similarly, the impaired F-box protein in CPE-109 can down-regulate the cytokinin biosynthetic repressor (*OsRR1*), resulting in up-regulation of cytokinin which subsequently leads to the CPE phenotype.

## Novel superior alleles for translational genomics: restructuring panicle architecture with CPE for developing high-yield future rice crop

The current study investigated a new panicle architectural trait, CPE, that exserted the panicle completely from the leaf sheath without elongating the uppermost internode significantly in rice. The article highlights the agronomic implications of the CPE trait and identifies key candidate genes and their associated functional mutations/alleles that influence this important trait, paving the way for future genetic improvement of rice. CPE is a recessive and quantitative complex trait controlled by two QTLs and its associated gene alleles. It is interesting to observe the significance of these induced mutations in genes with differential regulatory control over CPE, distinguishing them from EUI, despite both being influenced by GA signaling. Further, the combination of these alleles in the SM background with no pleiotropy indicates that CPE-109 can be directly introduced in the marker-assisted varietal trial for developing an improved SM with CPE phenotypes to impart a higher grain yield in rice. The functionally relevant mutations and superior alleles delineated in the genes can serve as targets for precise base editing by CRISPR (clustered regularly interspaced palindromic repeats) in several improved mega rice varieties to augment their yield advantage by the CPE trait (Fig. 1D). The identified KASP (Kompetitive allele-specific PCR) markers tightly linked to the QTLs and gene alleles with high PVE (phenotypic variance explained) for CPE have significance in rapid introgression of CPE into the popular cultivars through marker-assisted breeding for developing high-yielding rice varieties (Fig. 1E).

The salient findings of the study on CPE appear to be highly beneficial for the hybrid rice industry, enhancing the efficiency of commercial hybrid seed production and emphasizing its value in rice trade and commerce. CMS lines are frequently being used as female parents in rice hybrid seed production; however, the IPE phenotype is a common concern in these lines. The yield of the F_1_ hybrid or the multiplication of the male sterile line clearly depends on the efficient pollination of the CMS line by the restorer and maintainer line, respectively, which is being compromised by the partially opened panicle of the CMS line. Tailoring the genomic background of conventional CMS line with the discovered superior CPE trait-associated mutations and gene alleles, by marker-assisted breeding and/or using precise genome editing, can solve the common but major issue of incomplete panicle exsertion in rice. The genetically tailored rice varieties, restructured with the CPE trait through translational genomic intervention (marker-assisted breeding and genome editing), will address two key agronomic problems simultaneously in rice. Firstly, they can enhance the grain yield by exposing the sheathed spikelet for pollination. Secondly, unlike EUI, these improved rice varieties will not face the risk of panicle breakage and neck blast disease due to a normal uppermost internode (Fig. 1F). The novel genetic and genomic insight, particularly regarding panicle architectural traits, will be useful in opening up new avenues for translational genomic research for developing future high-yield hybrid rice that can contribute to future food security.
